# Suppression of anchorage-independent growth after gene transfection.

**DOI:** 10.1038/bjc.1993.323

**Published:** 1993-08

**Authors:** D. J. Winterbourne, S. Thomas, J. Hermon-Taylor

**Affiliations:** Department of Surgery, St George's Hospital Medical School, London, UK.

## Abstract

**Images:**


Br. J. Cancer (1993), 68, 251-258 © Macmillan Press Ltd., 1993

### Suppression of anchorage-independent growth after gene transfection

D.J. Winterbourne, S. Thomas & J. Hermon-Taylor

*Department of Surgery, St George's Hospital Medical School, Cranmer Terrace, London SW17 0RE, UK.*

**Summary** A novel procedure for isolating anchorage-dependent cells has been developed. It involves negative selection of cells growing in suspension followed by clonal replica screening for anchorage-dependent growth. Cells which have regained anchorage-dependent growth have been isolated from a library of the Chinese hamster ovary cell line, CHO-K1, transfected with pSV2neo and human genomic DNA. One anchorage-dependent clone, 1042AC, has been studied in detail. Anchorage-dependent growth of 1042AC is stable when cultured as adherent monolayers, but revertants appear rapidly when cultured in suspension. Suppression is unlikely to be due to loss or mutation of hamster genes conferring anchorage-independent growth as hybrids between 1042AC and CHO-K1 have the suppressed phenotype of 1042AC. Furthermore, a population of cells obtained from the hybrid by selecting for revertants to anchorage-independent growth showed selective loss of the transgenome derived from 1042AC. The growth suppression was not due to transfection of the human Krev-1 gene, which has previously been shown to restore anchorage-dependent growth, nor was there any evidence of alteration in the endogenous hamster Krev-1 gene. However, evidence for a human gene being responsible for the suppressed phenotype has not been obtained yet.

By comparison with the extensive knowledge of growth factors and their signal transducing pathways, the regulatory mechanisms of growth inhibition are poorly understood. Much of the information for such inhibitory mechanisms has come from studies of the tumour suppressor genes, whose functional loss may occur during neoplastic development (reviewed by Marshall, 1991). An alternative approach is to attempt to identify growth suppressor genes directly by phenotypic selection after gene transfection. Although tumour suppressor genes may have various functions, particularly in control of development, evidence that some can directly inhibit cell growth has been obtained for the retinoblastoma gene product (Huang *et al.*, 1988; Bookstein *et al.*, 1990; Madreperla *et al.*, 1991) and for p53 (Baker *et al.*, 1990; Diller *et al.*, 1990; Mercer *et al.*, 1990; Michalovitz *et al.*, 1990). Re-introduction of such genes may result in terminal arrest of cell growth (Huang *et al.*, 1988; Baker *et al.*, 1990; Diller *et al.*, 1990) requiring conditional expression of the transfected gene to allow development of stably transfected cell lines (Mercer *et al.*, 1990;Michalovitz *et al.*, 1990). It is unlikely that such genes, centrally involved in cell cycle control, can be isolated by transfection of unmodified DNA. However, for genes that conditionally arrest growth, this approach should be successful.

Assays for uncontrolled growth provide a simple, direct method of selecting for transformed cells and have been used to isolate activated oncogenes in DNA from human tumours. The converse of this approach, searching for genes that specifically suppress the transformed growth of cells, has been inadequately explored due to the inherent difficulty in isolating cells with a growth disadvantage. Negative selection procedures are usually inefficient, requiring combination with other selection or screening procedures (Noda, 1990). Despite these difficulties, human DNA capable of suppressing transformed phenotypes has been successfully isolated in a few cases (Schäfer *et al.*, 1988; Noda *et al.*, 1989; Eiden *et al.*, 1991). The best characterised gene, Krev-1, was present in only one of a series of flat revertants isolated in this way (Noda *et al.*, 1989), indicating that a number of genes may be involved in suppression of the transformed state.

We have chosen to investigate the mechanism which restricts cell growth in suspension. Cell transformation has been known for many years to result in loss of the normal requirement for attachment and spreading before cell division can occur (Stoker *et al.*, 1968) and the ability to grow in suspension is observed frequently in malignant cells (Shin *et al.*, 1975). However, despite the widespread use of this culture assay the relationship between anchorage and growth remains unclear. Although some growth factors can induce anchorage-independent growth, studies with somatic cell hybrids indicate that the phenotype is also regulated by growth suppressor genes (Marshall *et al.*, 1982; Islam *et al.*, 1989; Koi *et al.*, 1989). We have used the Chinese hamster ovary cell line (CHO-K1) which has been the subject of extensive genetic analysis and grows efficiently in suspension (Thompson, 1979). This report describes the development of an efficient negative selection procedure for cells whose growth in suspension has been arrested, and a novel clonal screening assay for anchorage-dependent growth. In combination, they allow isolation of cells solely on the basis of anchorage dependency. Using these procedures we have isolated variants of CHO-K1 that have substantially lost the ability to grow in suspension after DNA transfection.

### Materials and methods

### *Cell culture and transfection*

CHO-K1 cells were routinely cultured in 'complete medium' (αMEM (ICN Flow, High Wycombe, UK) with 10% (v/v) added newborn bovine serum without antibiotics) on 90 mm tissue culture dishes (Falcon 3003, Becton Dickinson). For negative selection and for precise determination of doubling times in stirred suspension, the CHO-K1 cells were grown in 500 ml culture vessels (Techne MCS stirrer, Techne (Cambridge) Ltd) stirred at 80 rpm in the same medium. Colony forming efficiency in 0.3% (w/v) agarose (Nicolson *et al.*, 1988) in complete medium was determined by counting colonies larger than 90 μm.

Electroporation tests were carried out in the presence of varying concentrations of the human genomic DNA mixed 1:1 with pSV2neo (Southern & Berg, 1982) or plasmids derived from it, using $3 \times 24\,\mu s$ pulses of $2.5\,kV\,cm^{-1}$ (Winterbourne *et al.*, 1988*b*). The cells were maintained at 20°C for 1 h before monitoring for DNA-dependent toxicity (Winterbourne *et al.*, 1988*b*). On the same day as these tests, cells were electroporated at $2 \times 10^7$ cells $ml^{-1}$ in the concentration of mixed plasmid and genomic DNA that gave 70% DNA-dependent toxicity. After 1 h at 20°C the majority of the cells were plated into 90 mm dishes for the main library selecting for geneticin resistance as described (Winterbourne *et al.*, 1988*b*). Four aliquots were also plated in duplicate 60 mm dishes for determination of stable transfection efficiency and survival from the electroporation. The colonies of geneticin resistant cells (containing on average about 2000 cells per colony) were collected by trypsinisation after thorough washing of the plates to remove non-adherent cells.

Correspondence: D.J. Winterbourne.
Received 16 December 1992; and in revised form 8 March 1993.

*Negative selection in stirred suspension culture*

Isolation of cells that do not grow under defined conditions may be carried out in a variety of ways, usually by killing cells that have replicated their DNA. We have adapted the H33258-enhanced killing by long wave u.v. light of cells that have incorporated 5-bromodeoxyuridine (Stetten *et al.*, 1977) to the efficient negative selection of cells growing in suspension (Winterbourne *et al.*, 1988*a*). Cells were suspended at about $10^5$ cells $ml^{-1}$ in medium containing 10 μm bromodeoxyuridine (Sigma, Poole, UK). After 3 days, H33258 (Sigma) was added to a final concentration of 1 μg $ml^{-1}$ 3 h before irradiation. The stirred suspension was irradiated for 30 s by a cylindrical arrangement of four lamps (Philips Actinic 09 long-wave u.v. lamps) concentric with the culture vessel, with a gap of 25 mm between the lamps and the wall of the vessel. The lamps have an emission spectrum which closely matches the excitation spectrum of H33258 and is negligible below 300 nm (manufacturer's data). The borosilicate glass of the culture vessel, which is only transparent above 300 nm, provided further protection from u.v. irradiation of unsubstituted DNA.

Cells were collected by centrifugation (300 *g* 5 min), washed once in phosphate buffered saline and resuspended in complete medium. The cells were cultured for one day in stirred suspension to allow cells to die, before being plated into tissue culture dishes. After 24 h to allow attachment of viable cells, the medium containing the dead cells was discarded, the dishes washed twice with phosphate buffered saline and fresh medium was added. Colonies of surviving cells grew up within 10 days. One plate was fixed and stained to estimate the survival frequency. The other plates were harvested for subsequent screening for clones having anchorage-dependent growth.

*Clonal screening assay for anchorage-dependent growth*

A replica plating method of screening individual clones for obligate anchorage-dependent growth was developed. Tissue culture treated microtest plates (Cat. No. 3596, Costar, Cambridge, MA) were seeded with one cell per well, on average. After growth to approximately 1000 cells per well, the plates were harvested using a multi-channel pipettor. Three replicas were made: the original master plate, a second tissue culture plastic plate, and a bacteriological grade plastic plate (Cat. No. 76-208-05, ICN Flow). The limited degree of attachment and spreading seen on some batches of bacteriological grade plastic was abolished by pre-incubation overnight at 37°C with 100 μl per well of 4 mg bovine serum albumin $ml^{-1}$. After 5–7 days growth, cells were quantified by staining. One tissue culture plastic plate was fixed and stained as described before (Winterbourne, 1986). Cells in the bacteriological grade plate were collected on No. 50 filter paper (Whatman Ltd, Maidstone, UK) using a 96 well dot blot apparatus (BioRad, Richmond, CA), washing the wells with 100 μl PBS to dislodge all cells. The cells were fixed on the filter with 3.7% formaldehyde in PBS, before removing from the apparatus. After drying, the filter was stained with Coomassie blue (Winterbourne, 1986). Tests with wild type CHO-K1 and an anchorage-dependent cell line showed the small number of cells seeded gave negligible signals, as did the anchorage-dependent cell line after 7 days growth in bacteriological grade plates.

The assay was scored by comparing the staining intensity of wells under adhesive conditions (tissue culture treated plastic) with the filter replica from wells under non-adhesive conditions (bacteriological grade plastic): an anchorage-dependent clone gave a signal on the stained plate, but not on the filter paper. Anchorage-independent cells, such as CHO-K1, gave signals under both conditions. No signal under either condition was either due to the chance absence of cells in the original Poisson distribution, or due to a cell that grew poorly under both conditions. Anchorage-dependent clones detected in this way were recovered from the master plate for further study.

As not all the wells containing cells in the screening assay will be of clonal origin, some anchorage-dependent clones may not be detected easily, due to overgrowth by a contaminating wild type cell or cells. To minimise the loss from this cause, we routinely picked cells from wells that were only marginally positive in the first screen. Such cells were then subjected to a second screen, using half a microtest plate. This resulted in the re-screening of about 30 sub-clones from each potentially positive well.

*Cell hybridisation*

Fusion of geneticin resistant cells with an anchorage-independent clone of mycophenolic acid resistant CHO-K1 transfected by electroporation with pSV2gpt (Mulligan & Berg, 1981) was induced by polyethylene glycol (Davidson & Gerald, 1977). Hybrids were selected in α-MEM containing 400 μg geneticin $ml^{-1}$, 25 μg mycophenolic acid $ml^{-1}$ (Gibco BRL), 2 μg aminopterin $ml^{-1}$ (Sigma), 250 μg xanthine $ml^{-1}$, 15 μg hypoxanthine $ml^{-1}$, 10 μg adenine $ml^{-1}$, 442 μg glutamine $ml^{-1}$, and supplemented with 10% dialysed newborn bovine serum.

*Preparation and analysis of DNA and RNA*

Human genomic DNA was prepared by proteinase K (Sigma) digestion of nuclei isolated from various human cell lines including GER, a pancreatic carcinoma line (Grant *et al.*, 1979). DNA was also prepared from white blood cells from a healthy human volunteer. The DNA preparations ran as smears on pulsed field gel electrophoresis with apparent size ranges of 50–800 kb (results not shown). Plasmids were prepared for transfection by alkaline lysis followed by purification on Sephacryl S1000 columns (Pharmacia LKB Biotechnology, Milton Keynes, UK).

Hybridisation analysis of 10 μg aliquots of restriction endonuclease digested DNA, fractionated on 0.7% agarose gels at 0.5 V $cm^{-1}$, were performed after alkaline transfer to Hybond N (Amersham International plc, UK). The blots were hybridised in a Hybritube 15 (GIBCO BRL, UK) with probes labelled with [$^{32}$P]CTP by the technique of Feinberg and Vogelstein (1984) either in 6 × SSC, 5 × Denhardt's solution, 0.5% SDS and 10% dextran sulphate with 100 μl $ml^{-1}$ sonicated salmon sperm DNA or in the buffer system of Church and Gilbert (1984). Probes, purified by electrophoresis, were the combined three small Pvu II fragments from pSV2neo, the 2 kb *Bam*H1 insert of Krev-1 (Kitayama *et al.*, 1989), the 1.3 kb insert of pRGAPDH-13 (Fort *et al.*, 1985) or human repetitive DNA prepared as described by Shih and Weinberg (1982). After washing at room temperature, blots were washed for 30 min at 65°C in 2 × SSC containing 0.1% SDS and then for 15 min at 65°C 0.1 × SSC, 0.1% SDS. For the human DNA probe, hybridisation buffer without Denhardt's solution contained 2 μg $ml^{-1}$ sonicated CHO-K1 DNA and the final high stringency wash was omitted. Before re-probing, blots were stripped by boiling for 15–30 min in 0.1% SDS. All washing and stripping steps were performed in the Hybritube.

RNA was prepared from sub-confluent plates of cells harvested by trypsinisation by the method described by Chomczynski and Sacchi (1987). Total RNA (20 μg) denatured by heating to 65°C for 15 min in electrophoresis buffer containing 1.8 M deionised formaldehyde and 50% deionised formamide was electrophoresed in a 1.5% agarose gel containing 0.7 M formaldehyde in 20 mM 3-(N-morpholino)propanesulphonic acid, 5 mM sodium acetate, 0.5 mM EDTA pH 7. RNA was blotted onto Hybond N+ membranes (Amersham International plc, UK) and fixed by u.v. irradiation before hybridisation analysis as above.

### Results

*Selection and screening of cells unable to grow in suspension*

The Chinese hamster ovary cell line, CHO-K1, grows readily both as an adherent monolayer and in stirred suspension culture. When wild type CHO-K1 cells, previously grown in suspension in bromodeoxyuridine under optimised conditions (Winterbourne *et al.*, 1988*a*) were exposed to light at 320 nm, the cells rapidly acquired lethal defects. Random mutations were not significantly increased by irradiation at this wavelength as lethal defects only occurred in cells that incorporated the thymidine analogue and was not seen when cells were grown in the absence of bromodeoxyuridine (Table I). Irradiation for 30 s was chosen for subsequent experiments.

Despite the highly efficient negative selection some survivors were seen when large numbers of cells were subjected to the procedure. Randomly chosen survivors were not anchorage-dependent in subsequent tests and appeared to be wild type CHO-K1 that had escaped the negative selection. Standard assays of anchorage-independency in viscous medium do not permit isolation of cells that fail to grow. To overcome this problem we developed a method of screening replicas of surviving clones for anchorage-dependent growth. Combination of the H33258-bromodeoxyuridine selection procedure on about $5 \times 10^7$ cells and the two pass clonal screening of subsequent survivors, detected no variants that had anchorage-dependent growth from wild type CHO-K1 or a subclone (Table II). Thus, spontaneous appearance of the anchorage-dependent growth phenotype in these cells seems to be very rare (less than 1 in 10 million cells).

### *Restoration of anchorage-dependent growth following transfection*

Seven libraries of CHO-K1 cells containing 20 to 100,000 independent clones bearing transfected DNA were prepared. Each library was created by pooling the geneticin resistant colonies recovered after transfecting $2 \times 10^7$ CHO-K1 cells with a neo containing plasmid and different sources of human DNA, including human tumour cell lines. Although specific tumour suppressor genes may be inactivated in individual tumours, it is probable that other genes will remain functional. The frequency of stable geneticin resistance observed during the preparation of these libraries was between 0.1 and 0.6% of the electroporated cells.

Cultures of each library were subjected to the negative selection procedure in stirred suspensions. As expected, the number of survivors were similar to those obtained with the untransfected parental cells (Table II). For each selection experiment, 200 to 300 independent survivors were subjected to the microtest screening assay. Cells from positive or indeterminate wells were recovered from the master plates and rescreened using half a plate for each clone (yielding about 30 wells with sub-clones). Only in experiments with two libraries did cells surviving negative selection remain positive in this second clonal anchorage-dependency screen: nine clones were isolated after transfecting with DNA from the GER human pancreatic carcinoma cell line and two with DNA from normal white blood cells. Examples of the second screen with the GER library, which gave clear positive results, are shown in Figure 1.

**Table I** Affect of incorporation of bromodeoxyuridine on survival of cells exposed to light

| *Irradiation time (s)* | *Survival frequency* Control | *10 μM BrdU* |
|---|---|---|
| 0 | 0.79 | 0.14 |
| 5 | 0.80 | $7.4 \times 10^{-5}$ |
| 15 | 0.99 | $1.1 \times 10^{-6}$ |
| 30 | 0.80 | $1.4 \times 10^{-6}$ |
| 60 | 0.88 | $0.9 \times 10^{-6}$ |
| 300 | 0.77 | $0.7 \times 10^{-6}$ |
| 600 | 0.62 | $0.5 \times 10^{-6}$ |
| 1800 | 0.74 | $0.6 \times 10^{-6}$ |

CHO-K1 cells were cultured in stirred suspensions in the presence or absence of 10 μM bromodeoxyuridine for 3 days. Both cultures, after 3 h incubation with 1 μg H33258 $ml^{-1}$, were irradiated at 320 nm. Cell viability was determined by clonal plating efficiency on tissue culture plastic, after various periods of irradiation.

**Table II** Application of the selection and screening procedure to wild-type CHO-K1

| | *Mass culture* | *Sub-clone* |
|---|---|---|
| *Negative-selection in suspension* | | |
| Initial cell number | $60 \times 10^6$ | $50 \times 10^6$ |
| Final cell number | $660 \times 10^6$ | $450 \times 10^6$ |
| Survivors | 440 | 1250 |
| *Screening for anchorage-dependent growth* | | |
| Number of clones screened | 296 | 244 |
| Initially 'positive' wells | 8 | 4 |
| Anchorage-dependent clones | 0 | 0 |

The number of wild-type CHO-K1 cells (both the original mass culture and a subclone) at the beginning and end of the period of growth in bromodeoxyuridine is shown, as are the number of cells surviving the exposure to light at 320 nm and subsequently growing as colonies on plastic. To avoid the possibility of missing positive wells in the screening for anchorage dependency, even weakly positive wells were picked in the first round. About 30 individual cells from each positive well were subsequently re-screened.

### *Growth characteristics of the anchorage-dependent cells*

Of the nine anchorage-dependent clones isolated after transfecting with GER DNA, the cell line designated 1042AC was selected for subsequent studies. The defective growth of 1042AC in stirred suspensions was confirmed (Figure 2). Doubling times, estimated from such experiments, were 100 h for 1042AC compared with 21 h for the wild type CHO-K1. Similarly, colony forming efficiency in 0.3% agarose was 8% for 1042AC compared with 61% for CHO-K1. Despite the five-fold reduction in growth rate in stirred suspension, the cells grew at similar rates when attached to tissue culture treated dishes (a doubling time of 18 h for 1042AC compared with 16 h for CHO-K1). Although morphology was not used as a criterion in the selection of the anchorage-dependent

**Figure 1** Second screen for anchorage-dependent growth. Replica microtest plates were prepared from random clones of the survivors from the negative selection after transfection with GER DNA and screened for anchorage-dependent growth. Cells from wells scoring positively in the first round were re-screened by seeding at a clonal density in half the wells of a microtest plate. Examples of this re-screen for four of the wells initially scoring positively are shown. **a**, and **e**, a false positive clone; **b**, and **f**, a clone with poor growth characteristics under both conditions; **c**, and **g**, **d**, and **h**, two anchorage-dependent clones. **a**,-**d**,: adherent culture. **e**,-**h**,: suspension culture.

1042AC cells, they had a more elongated fibroblastic morphology with a greater tendency to form lateral alignments than the randomly oriented, compact, parental CHO-K1 (Figure 3a and b).

Anchorage-dependent cells tended to clump during their slow growth in stirred suspension culture. When the cultures were trypsinised and reseeded at a lower density, it was found that the subsequent growth in stirred supspension was dramatically increased to a rate similar to the wild type cells (20 h doubling time). This reproducible effect could be due to the loss or inactivation of a transfected suppressor gene in a small number of 1042AC cells, with subsequent selection of these revertants by their growth advantage in suspension culture. It was possible to obtain a good simulation of the observed results assuming reversion occurred spontaneously at a frequency of $10^{-5}$, using a simple model of exponential growth (not shown). The population of revertant cells obtained in this way was designated 1052Rev, these cells retained their ability to grow in suspension after many passages as adherent monolayers. The morphology of 1052Rev (Figure 3c) more closely resembled that of the wild type CHO-K1 than the anchorage-dependent 1042AC cells.

The anchorage-dependent phenotype of 1042AC cells cultured continuously under non-selective conditions as adherent monolayers, was moderately stable. When 1042AC cultured for 28 passages as an adherent monolayer, was assayed in stirred suspension the doubling time was approximately 64 h, indicating only partial loss of the suppressed anchorage-independent growth phenotype. This stirred suspension culture showed the same stepwise shift to faster growth (26 h doubling time) when trypsinised and reseeded at a lower density in suspension (not shown).

### *DNA and RNA analysis of transfected cell lines*

Of the nine anchorage-dependent clones isolated after transfection with GER DNA, six appeared to have the neo gene integrated at the same site (Figure 4a), indicating that these may be independent isolations of cells for a single transfection event. Analysis of randomly selected clones showed restriction fragment length polymorphisms, indicating random integration of the neo vector (not shown). The suppression of anchorage-independent growth was not due to transfection of the human Krev-1 gene, as only the endogenous hamster gene was detected (Figure 4b). Re-probing the blots with human repetitive DNA, gave only weak signals for the presence of human DNA in the clones (Figure 4c). These data and the presence of only single copies of pSV2neo is consistent with integration and expression of much smaller amounts of DNA when transfection is induced by electroporation rather than by precipitation techniques.

The analysis of DNA isolated from late passage 1042AC, which had partially reverted to anchorage-independent growth, and from the revertant 1052Rev appeared to be similar to early passage 1042AC, although the signals on this blot were weak (Figure 4). In other experiments, no differences were detected on southern blots of *Bam*H1, *Eco*RI and *Hind*III digested DNA isolated from 1042AC and the revertant 1052Rev when probed with neo and Krev-1 (not shown). All blots were also probed with human repetitive DNA, but gave only weak signals which showed no reproducible differences between CHO-K1 and any of the cell lines derived from it. Analysis of RNA showed that the revertant still expressed the neo gene (Figure 5a) and that there was no significant difference in the level of expression of the Krev-1 gene between 1042AC, 1052Rev and the wild type cells (Figure 5b). The amount of RNA in each lane was similar as shown by the signal for GAPDH (Figure 5c).

### *Dominance of anchorage-dependent phenotype*

CHO-K1 stably transfected with pSV2gpt was fused with 1042AC and a hybrid clone was obtained by selection with mycophenolic acid and geneticin. When cultured in stirred suspension, the hybrid showed the same phenotype as

**Figure 2** Growth of CHO-K1 and an anchorage-dependent transfectant in stirred suspension culture. CHO-K1 cells (circles) and the anchorage-dependent clone 1042AC (triangles) were seeded at $10^5$ cells ml$^{-1}$ of complete medium and cultured under standard conditions. At intervals aliquots were removed, the cells collected by centrifugation, trypsinised and counted. After 14 days in culture, 1042AC cells were collected by centrifugation, trypsinised and re-seeded at a lower density (arrow).

**Figure 3** Morphology of cells. Phase contrast micrographs of colonies of **a**, wild type CHO-K1, **b**, the anchorage-dependent transfectant 1042AC and **c**, the derivative 1052Rev growing on tissue culture plastic. Bar, 250 μm.

**Figure 4** Hybridisation analysis of *Eco*RI digested genomic DNA from anchorage-dependent clones. The blot was hybridised with probes for **a**, neo, **b**, Krev-1 and **c**, human repetitive DNA. Lanes 1–9: DNA from nine independently isolated anchorage-dependent clones; DNA in lane 2 was isolated from the cell line called 1042AC. Lane 10: 1042AC after 25 passages in continuous culture as an adherent monolayer. Lane 11: 1052Rev. Lane 12: wild type CHO-K1. The last three lanes contain standards of 3, 9 and 27 pg *Eco*RI digested pSV2neo with 3, 9 and 27 ng *Eco*RI digested human DNA.

1042AC i.e. suppressed growth over the first two weeks followed by a stepwise shift to faster growth after trypsinisation and reseeding at lower density (Figure 6). The subsequent revertant to anchorage-independent growth obtained from this hybrid still retained the mycophenolic acid resistance of the wild type parent, but the majority of the cells from the revertant population had lost their resistance to geneticin conferred by 1042AC (relative colony forming efficiency in geneticin 17%). By contrast 1052Rev, the anchorage-independent revertant derived directly from 1042AC, retained the geneticin resistance (relative colony forming efficiency in geneticin 83%).

Probing Southern blots for the drug-resistance marker genes confirmed that the fusion product with suppressed anchorage-independent growth was a hybrid (Figure 7, lanes 4–6). In addition, this analysis showed a selective reduction in the signal for the pSV2neo marker in DNA from the revertant population derived from the hybrid (Figure 7, lanes 6 and 7), in agreement with the loss of geneticin resistance described above. This result suggests that reversion in the suppressed hybrid may occur by simultaneous loss of a co-transfected growth suppressor gene and the pSV2neo marker.

**Figure 5** Analysis of $neo^r$ and Krev-1 expression. Total RNA transferred to a Hybond N+ membrane was hybridised with **a**, neo, **b**, Krev-1 and **c**, glyceraldehyde 3-phosphate dehydrogenase gene probes. Lane 1 – CHO-K1; lane 2 – 1042AC; lane 3 – 1052Rev.

**Figure 6** Stirred suspension culture growth of the hybrid between 1042AC and anchorage-independent pSV2gpt transfected CHO-K1 cells. Hybrid cells were seeded at $10^5$ cells $ml^{-1}$ of complete medium and cultured as described in Figure 2.

### Discussion

The high efficiency of our combined selection and screening methods was illustrated by the low frequency ($<10^{-8}$) at which anchorage-dependent clones were observed. This contrasts with the large number of survivors observed by others using a different negative selection protocol and calcium phosphate transfected cells (Padmanabhan *et al.*, 1987). The unconditional inhibition of growth in that study appeared to be due to transfected repetitive DNA (Padmanabhan *et al.*, 1987) and is unlikely to explain the results described here. The development of efficient procedures for isolation of

**Figure 7** Hyrbidisation analysis of *Eco*RI digested genomic DNA from 1042AC, the anchorage-dependent hybrid and its revertants. The blot was hybridised with a probe of linearised pSV2neo which detects both pSV2neo and pSV2gpt. (1) wild type CHO-K1, (2 and 5) 1042AC, (3) 1052Rev, (4) mycophenolic acid resistant CHO-K1, (6) hybrid, (7) hybrid revertant. The last three lanes contain standards as described in Figure 4.

anchorage-dependent cells based only on their growth properties should be of use to others, avoiding the need to link growth with phenotypes such as morphology or lectin-agglutinability.

The rare cells with restored anchorage-dependent growth isolated after DNA transfection may have arisen by transfection of a human gene that suppresses anchorage-independent growth. However, we have been unable to demonstrate this so far and we cannot rule out the possibility that the low frequency of suppression may have arisen by some other mechanism not specifically involving a human suppressor gene. Thus, transfected DNA may have resulted either in reduced expression of an endogenous hamster gene required for anchorage-independent growth or increased expression of one that suppresses such growth. It is unlikely that anchorage-dependent variants preexisting in the population of CHO-K1 were isolated, as seven out of nine complete selection and screening experiments yielded no suppressed cells. Five of the seven experiments that failed to yield suppressed cells were obtained with cells that bore transfected DNA (at least the selectable plasmid DNA). Therefore the results are also unlikely to be due to a non-specific effect of transfection or an effect of the selection pressure imposed by growth from low density during the isolation of the geneticin resistant libraries.

An example in which similar experiments resulted in reduced expression of an endogenous gene (the fos transformation effector gene) was reported by Kho and Zarbl (1992). Re-introduction of the cloned fte-1 gene, the endogenous copy of which had been disrupted by the initial transfection event, restored the transformed phenotype (Kho & Zarbl, 1992). In contrast, the suppressed phenotype of 1042AC cells in the present study was dominant in somatic cell hybrids (Figure 6). Furthermore, revertants that regained the ability to grow in suspension retained the inserted pSV2neo DNA and presumably also the disruption of the endogenous sequence at this site. Although these results indicate that suppression in 1042AC is unlikely to be due to inactivation of hamster genes conferring anchorage-independent growth, they do not exclude the possibility that damage to the recipient genome may be responsible for the suppressed phenotype. However, if this is the case, the plasmid is a marker that may allow cloning of the relevant gene.

The phenotypic dominance and the subsequent loss of suppression under selection pressure for anchorage-independence would be consistent with the acquisition and subsequent loss of exogenous genes. However, using repetitive-DNA probes, we have been unable to show convincingly the presence of human DNA in the suppressed cell line. The inability to detect human sequences in cells that were subsequently shown to bear a transfected human gene has been reported previously (Pinney *et al.*, 1988). Although repetitive sequences are widely dispersed throughout the human genome, the distribution is not uniform (Schmid & Jelinek, 1982; McCombie *et al.*, 1992). Also, there is considerable homology between human and rodent Alu sequences and variation between individual members of the human Alu family (Schmid & Jelinek, 1982). The combination of these factors may have contributed to our failure unequivocally to detect human DNA in 1042AC at the present stage.

The suppression of anchorage-independent growth was moderately stable under non-selective conditions and was lost only when suppressed cells were continuously cultured in suspension. Selection of revertants to anchorage-independent growth from the hybrid resulted in concomitant loss of geneticin, but not mycophenolic acid, resistance. As whole chromosome loss is a frequent event in hybrid cells and co-transfected DNA may become physically linked during integration (Perucho *et al.*, 1980) revertants from the suppressed hybrid may have arisen by loss of a chromosome containing both the geneticin resistance marker and a putative suppressing gene. We are attempting to clone the transgenome from a genomic library of 1042AC DNA to test this possibility.

Human genes have been isolated in two studies in which the transformed phenotype induced by activated *ras* was suppressed by DNA transfection (Schäfer *et al.*, 1988; Kitayama *et al.*, 1989; Noda *et al.*, 1989). One of these genes, Krev-1, has been shown to share homology with, but have opposing actions to *ras* (Zhang *et al.*, 1990). Our results show that anchorage-dependence induced in the suppressed CHO-K1 cells is not due to a transfected human Krev-1 gene, nor is expression of the endogenous hamster Krev-1 gene modified. We have also found that direct transfection of Krev-1 does not efficiently suppress anchorage-independent growth of CHO-K1 (unpublished observations).

The second *ras*-transformation suppressor gene was detected as an 18 kb restriction fragment that suppressed anchorage-independent growth of EJ-*ras*-transformed rat fibroblasts. It is unlikely to be responsible for the results reported here as this gene was detectable with repetitive human DNA probes (Schäfer *et al.*, 1988). In another study, selecting against growth of spontaneously transformed Chinese hamster cells in low serum, Schäfer *et al.*( 1991) have isolated another human DNA marker indirectly associated with tumour suppression. In neither case has the identity of these suppressor genes yet been reported.

Recently, Eiden *et al.* (1991) used direct microscopic examination of colony morphology to isolate a cDNA that suppressed the chemically transformed phenotype of BHK cells. The cDNA was found to be the partially processed human vimentin gene. However, no differences in the expression or size of vimentin proteins could be detected between the transformed or suppressed cells, leading the authors to suggest that the original chemical transformation may have resulted from small deletions or point mutations in the BHK vimentin gene (Eiden *et al.*, 1991). Significantly, Chan *et al.* (1989) have shown that increased phosphorylation of vimentin was one of a small number of alterations in protein phosphorylation that correlated with the reversion of the transformed phenotype of CHO-K1 cells induced by cyclic AMP.

The transient effects of cyclic AMP on CHO-K1 morphology and anchorage-independent growth (Hsie & Puck, 1971; Puck, 1977) are remarkably similar to the stable effects obtained here after DNA transfection into the same cell line. The ability of cyclic AMP to increase the phosphorylation of vimentin and other proteins (Chan *et al.*, 1989) indicates that any defect in the organisation of the intermediate filament protein in CHO-K1 is unlikely to reside in the vimentin gene itself, as may be the case in the chemically transformed BHK cells (Eiden *et al.*, 1991). Instead, it would appear to reside in control of cyclic AMP-dependent phosphorylations. These results suggest that one possible mechanism for the suppression of anchorge-independent growth in 1042AC cells may be correction of defects in the regulation of cyclic AMP-dependent phosphorylations. This possibility will be the subject of further investigation.

This work was supported by the Mike Stone Cancer Research Fund. We thank Miss M.A. Stebbing, MD, FRCS for help in preparing some of the CHO-K1 libraries.

### References

BAKER, S.J., MARKOWITZ, S., FEARON, E.R., WILLSON, J.K. & VOGELSTEIN, B. (1990). Suppression of human colorectal carcinoma cell growth by wild-type p53. *Science*, **249**, 912–915.

BOOKSTEIN, R., SHEW, J.Y., CHEN, P.L., SCULLY, P. & LEE, W.H. (1990). Suppression of tumorigenicity of human prostate carcinoma cells by replacing a mutated RB gene. *Science*, **247**, 712–715.

CHAN, D., GOATE, A. & PUCK, T.T. (1989). Involvement of vimentin in the reverse transformation reaction. *Proc. Natl Acad. Sci. USA*, **86**, 2747–2751.

CHOMCZYNSKI, P. & SACCHI, N. (1987). Single-step method of RNA isolation by acid guanidinium thiocyanate-phenol-chloroform extraction. *Anal. Biochem.*, **162**, 156–159.

CHURCH, G.M. & GILBERT, W. (1984). Genomic sequencing. *Proc. Natl Acad. Sci. USA*, **81**, 1991–1995.

DAVIDSON, R.L. & GERALD, P.I. (1977). Induction of mammalian somatic cell hybridization by polyethylene glycol. *Methods Cell Biol.*, **15**, 325–338.

DILLER, L., KASSEL, J., NELSON, C.E., GRYKA, M.A., LITWAK, G., GEBHARDT, M., BRESSAC, B., OZTURK, M., BAKER, S.J., VOGELSTEIN, B. & FRIEND, S.H. (1990). p53 functions as a cell cycle control protein in osteosarcomas. *Mol. Cell Biol.*, **10**, 5772–5781.

EIDEN, M.V., MACARTHUR, L. & OKAYAMA, H. (1991). Suppression of the chemically transformed phenotype of BHK cells by a human cDNA. *Mol. Cell Biol.*, **11**, 5321–5329.

FEINBERG, A. & VOGELSTEIN, B. (1984). A technique for radiolabelling DNA restriction nuclease fragments to high specific activity – Addendum. *Anal. Biochem.*, **137**, 266–267.

FORT, P., MARTY, L., PIECHACZYK, M., EL SABROUTY, S., DANI, C., JEANTEUR, P. & BLANCHARD, J.M. (1985). Various rat adult tissues express only one major mRNA species from the glyceraldehyde-3-phosphate-dehydrogenase multigenic family. *Nucleic Acids Res.*, **13**, 1431–1442.

GRANT, A.G., DUKE, D. & HERMON-TAYLOR, J. (1979). Establishment and characterization of primary human pancreatic carcinoma in continuous cell culture and in nude mice. *Br. J. Cancer*, **39**, 143–151.

HSIE, A.W. & PUCK, T.T. (1971). Morphological transformation of Chinese hamster cells by dibutyryl adenosine cyclic 3':5'-monophosphate and testosterone. *Proc. Natl Acad. Sci. USA*, **68**, 358–361.

HUANG, H.J., YEE, J.K., SHEW, J.Y., CHEN, P.L., BOOKSTEIN, R., FRIEDMANN, T., LEE, E.Y. & LEE, W.H. (1988). Suppression of the neoplastic phenotype by replacement of the RB gene in human cancer cells. *Science*, **242**, 1563–1566.

ISLAM, M.Q., SZPIRER, J., SZPIRER, C., ISLAM, K., DASNOY, J.F. & LEVAN, G. (1989). A gene for the suppression of anchorage independence is located in rat chromosome 5 bands q22–23, and the rat alpha-interferon locus maps at the same region. *J. Cell Sci.*, **92**, 147–162.

KHO, C.J. & ZARBL, H. (1992). Fte-1, a v-fos transformation effector gene, encodes the mammalian homologue of a yeast gene involved in protein import into mitochondria. *Proc. Natl Acad. Sci. USA*, **89**, 2200–2204.

KITAYAMA, H., SUGIMOTO, Y., MATSUZAKI, T., IKAWA, Y. & NODA, M. (1989). A ras-related gene with transformation suppressor activity. *Cell*, **56**, 77–84.

KOI, M., AFSHARI, C.A., ANNAB, L.A. & BARRETT, J.C. (1989). Role of tumour-suppressor gene in the negative control of anchorage-independent growth of Syrian hamster cells. *Proc. Natl Acad. Sci. USA*, **86**, 8773–8777.

MADREPERLA, S.A., WHITTUM-HUDSON, J.A., PRENDERGAST, R.A., CHEN, P.L. & LEE, W.H. (1991). Intraocular tumor suppression of retinoblastoma gene-reconstituted retinoblastoma cells. *Cancer Res.*, **51**, 6381–6384.

MARSHALL, C.J. (1991). Tumor suppressor genes. *Cell*, **64**, 313–326.

MARSHALL, C.J., KITCHIN, R.M. & SAGER, R. (1982). Genetic analysis of tumourigenesis: XII. Genetic control of the anchorage requirement in CHEF cells. *Somatic Cell Genet.*, **8**, 709–721.

MCCOMBIE, W.R., MARTIN-GALLARDO, A., GOCAYNE, J.D., FITZGERALD, M., DUBNICK, M., KELLEY, J.M., CASTILLA, L., LIU, L.I., WALLACE, S., TRAPP, S., TAGLE, D., WHALEY, W.L., CHENG, S., GUSELLA, J., FRISCHAUF, A.-M., POUSTKA, A., LEHRACH, H., COLLINS, F.S., KERLAVAGE, A.R., FIELDS, C. & VENTER, J.C. (1992). Expressed genes, Alu repeats and polymorphisms in cosmids sequenced from chromosome 4p16.3. *Nature Genetics*, **1**, 348–353.

MERCER, W.E., SHIELDS, M.T., AMIN, M., SAUVE, G.J., APPELLA, E., ROMANO, J.W. & ULLRICH, S.J. (1990). Negative growth regulation in a glioblastoma tumor cell line that conditionally expresses human wild-type p53. *Proc. Natl Acad. Sci. USA*, **87**, 6166–6170.

MICHALOVITZ, D., HALEVY, O. & OREN, M. (1990). Conditonal inhibition of transformation and of cell proliferation by a temperature-sensitive mutant of p53. *Cell*, **62**, 671–680.

MULLIGAN, R.C. & BERG, P. (1981). Selection for animal cells that express the E. coli gene coding for xanthine-guanine phosphoribosyltransferase. *Proc. Natl Acad. Sci. USA*, **78**, 2072–2076.

NICOLSON, G.L., LEMBO, T.M. & WELCH, D.R. (1988). Growth of rat mammary adenocarcinoma cells in semisolid clonogenic medium not correlated with spontaneous metastatic behaviour: heterogeneity in the metastatic, antigenic, enzymatic and drug sensitivity properties of cells from different sized colonies. *Cancer Res.*, **48**, 399–404.

NODA, M. (1990). Expression cloning of tumor suppressor genes: a guide for optimists. *Molecular Carcinogenesis*, **3**, 251–253.

NODA, M., KITAYAMA, H., MATSUZAKI, T., SUGIMOTO, Y., OKAYAMA, H., BASSIN, R.H. & IKAWA, Y. (1989). Detection of genes with a potential for suppressing the transformed phenotype associated with activated ras genes. *Proc. Natl Acad. Sci. USA*, **86**, 162–166.

PADMANABHAN, R., HOWARD, T.H. & HOWARD, B.H. (1987). Specific growth inhibitory sequences in genomic DNA from quiescent human embryo fibroblasts. *Mol. Cell Biol.*, **7**, 1894–1899.

PERUCHO, M., HANAHAN, D. & WIGLER, M. (1980). Genetic and physical linkage of exogenous sequences in transformed cells. *Cell*, **22**, 309–317.

PINNEY, D.F., PEARSON-WHITE, S.H., KONIECZNY, S.F., LATHAM, K.F. & EMERSON, C.P. Jr. (1988). Myogenic lineage determination and differentiation: evidence for a regulatory pathway. *Cell*, **53**, 781–793.

PUCK, T.T. (1977). Cyclic AMP, the microtubule-microfilament system and cancer. *Proc. Natl Acad. Sci. USA*, **74**, 4491–4495.

SCHÄFER, R., IYER, J., ITEN, E. & NIRKKO, A.C. (1988). Partial reversion of the transformed phenotype in HRAS-transfected tumorigenic cells by transfer of a human gene. *Proc. Natl Acad. Sci. USA*, **85**, 1590–1594.

SCHÄFER, R., NIRKKO, A.C., AMBÜHL, P.M., GRZESCHIK, K.H. & SCHWARTE-WALDHOFF, I. (1991). Evidence for human DNA-mediated transfer of the suppressed phenotype into malignant Chinese hamster cells. *Oncogene*, **6**, 2221–2228.

SCHMID, C.W. & JELINEK, W.R. (1982). The Alu family of dispersed repeats. *Science*, **216**, 1065–1070.

SHIH, C. & WEINBERG, R.A. (1982). Isolation of a transforming sequence from a human bladder carcinoma cell line. *Cell*, **29**, 161–169.

SHIN, S., FREEDMAN, V.H., RISER, R. & POLLACK, R. (1975). Tumourigenicity of virus-transformed cells in nude mice is correlated specifically with anchorage independent growth *in vitro*. *Proc. Natl Acad. Sci. USA*, **72**, 4435–4439.

SOUTHERN, P.J. & BERG, P. (1982). Transformation of mammalian cells to antibiotic resistance with a bacterial gene under control of the SV40 early region promoter. *J. Mol. Appl. Genet.*, **1**, 327–341.

STETTEN, G., DAVIDSON, R.L. & LATT, S.A. (1977). 33258 Hoechst enhances the selectivity of the bromodeoxyuridine-light method of isolating conditional lethal mutants. *Exp. Cell Res.*, **108**, 447–452.

STOKER, M.G.P., O'NEILL, C., BERRYMAN, S. & WAXMAN, V. (1968). Anchorage and growth regulation in normal and virus transformed cells. *Int. J. Cancer.*, **3**, 683–689.

THOMPSON, L.H. (1979). Mutant isolation. *Methods Enzymol.*, **58**, 308–322.

WINTERBOURNE, D.J. (1986). Cell growth determined by a dye-binding protein assay. *Biochem. Soc. Trans.*, **14**, 1179.

WINTERBOURNE, D.J., THOMAS, S., STEBBING, A. & HERMON-TAYLOR, J. (1988*a*). A procedure for selection of cells with suppressed growth in suspension after gene transfection. *Biochem. Soc. Trans.*, **16**, 1018.

WINTERBOURNE, D.J., THOMAS, S., HERMON-TAYLOR, J., HUSSAIN, I. & JOHNSTONE, A.P. (1988*b*). Electric shock mediated transfection of cells: characterization and optimization of electrical parameters. Biochem. J., **251**, 427–434.

ZHANG, K., NODA, M., VASS, W.C., PAPAGEORGE, A.G. & LOWY, D.R. (1990). Identification of small clusters of divergent amino acids that mediate the opposing effects of ras and Krev-1. *Science*, **249**, 162–165.


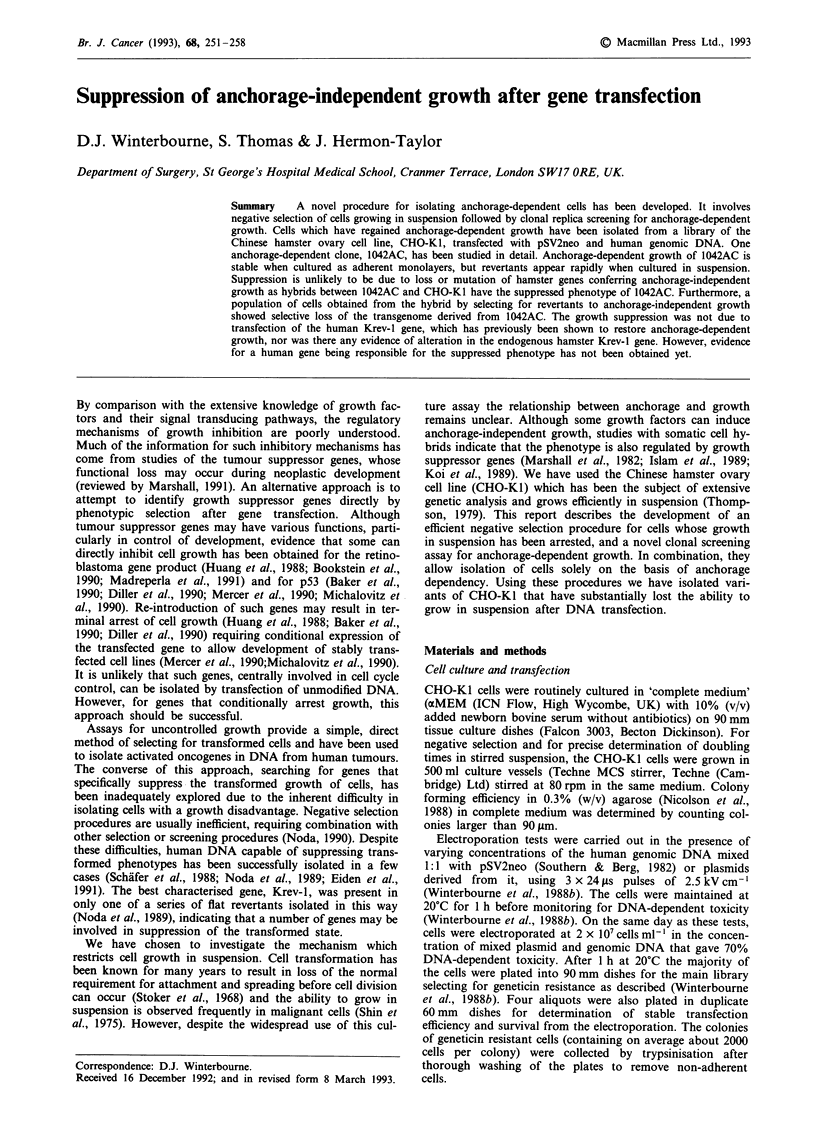

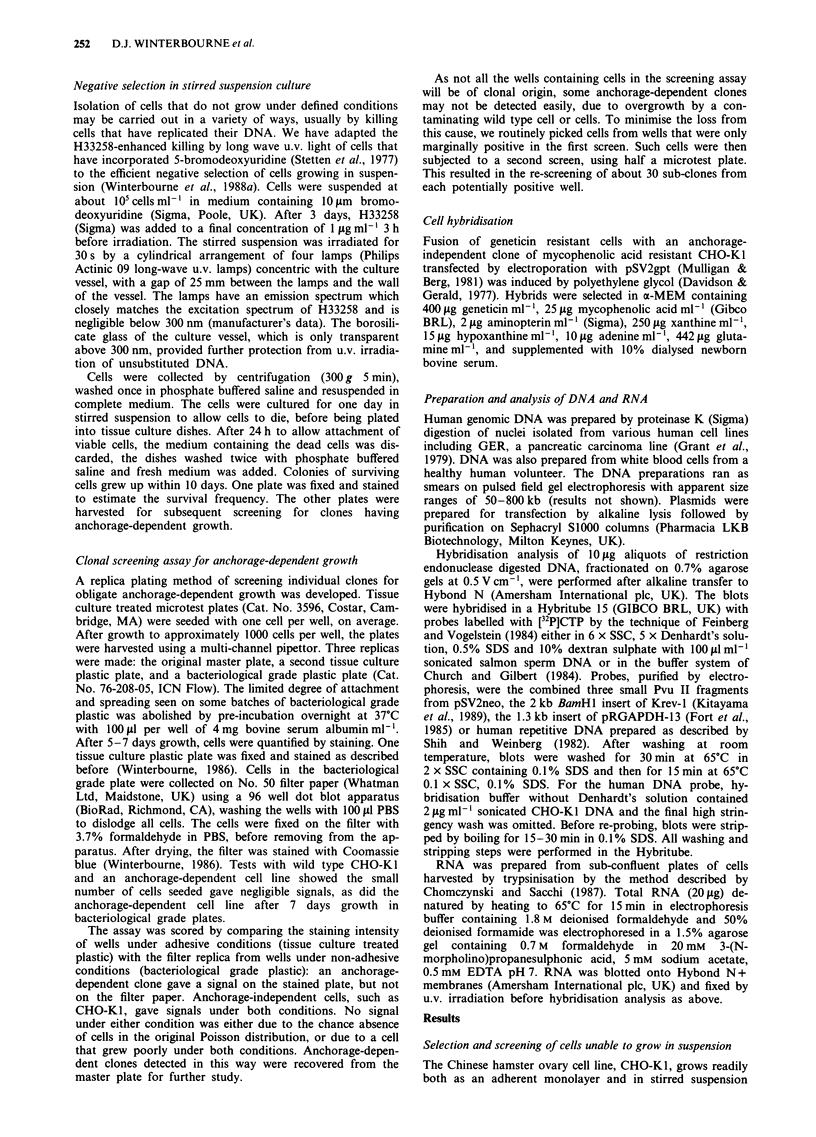

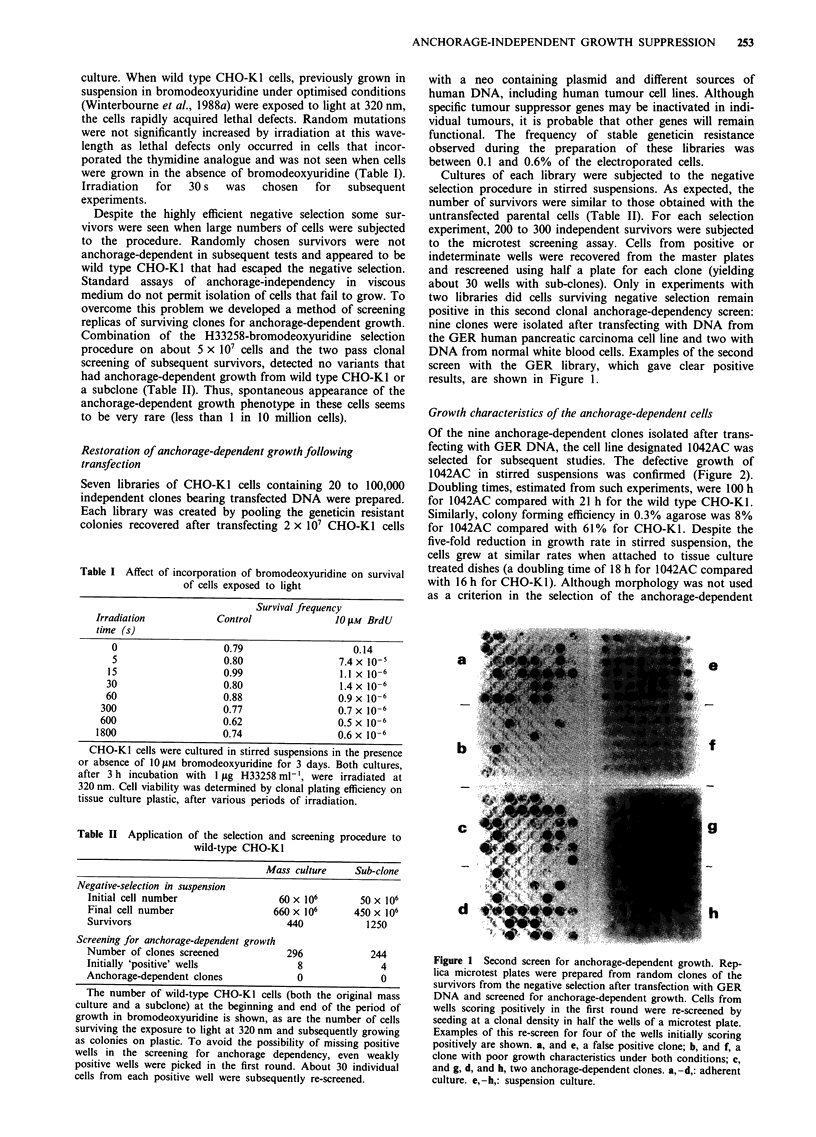

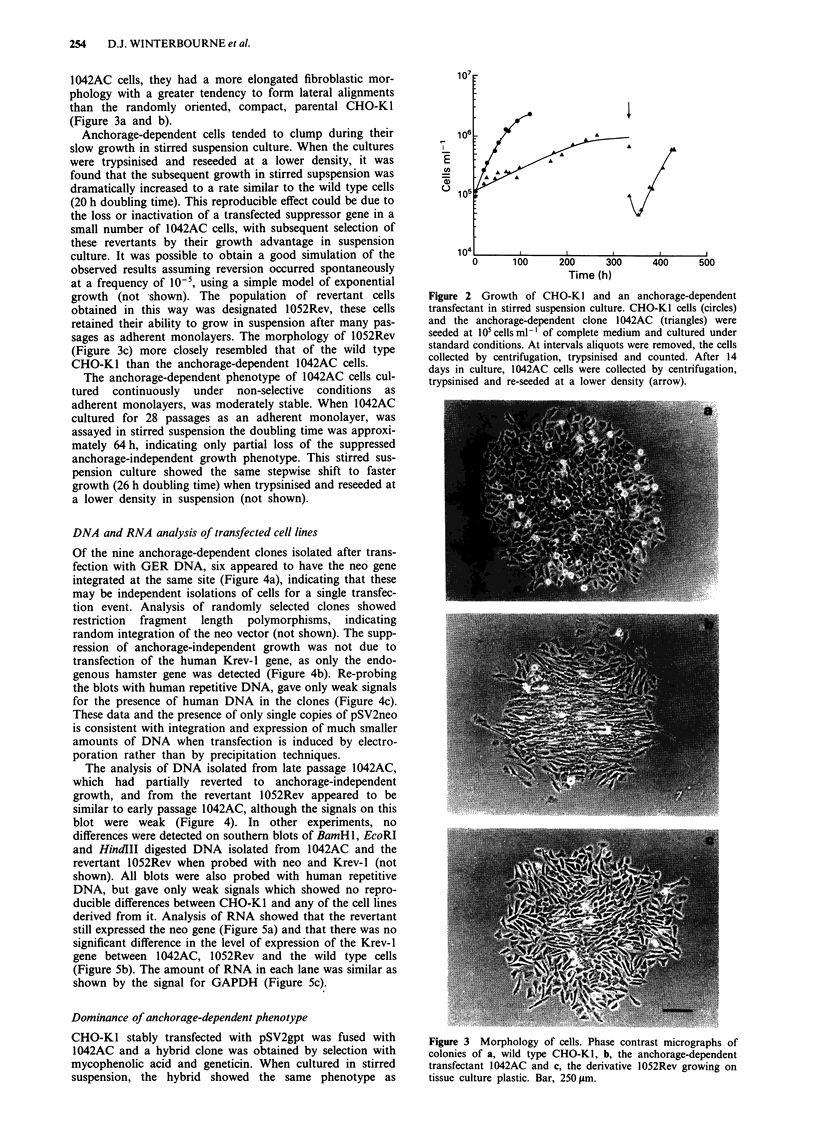

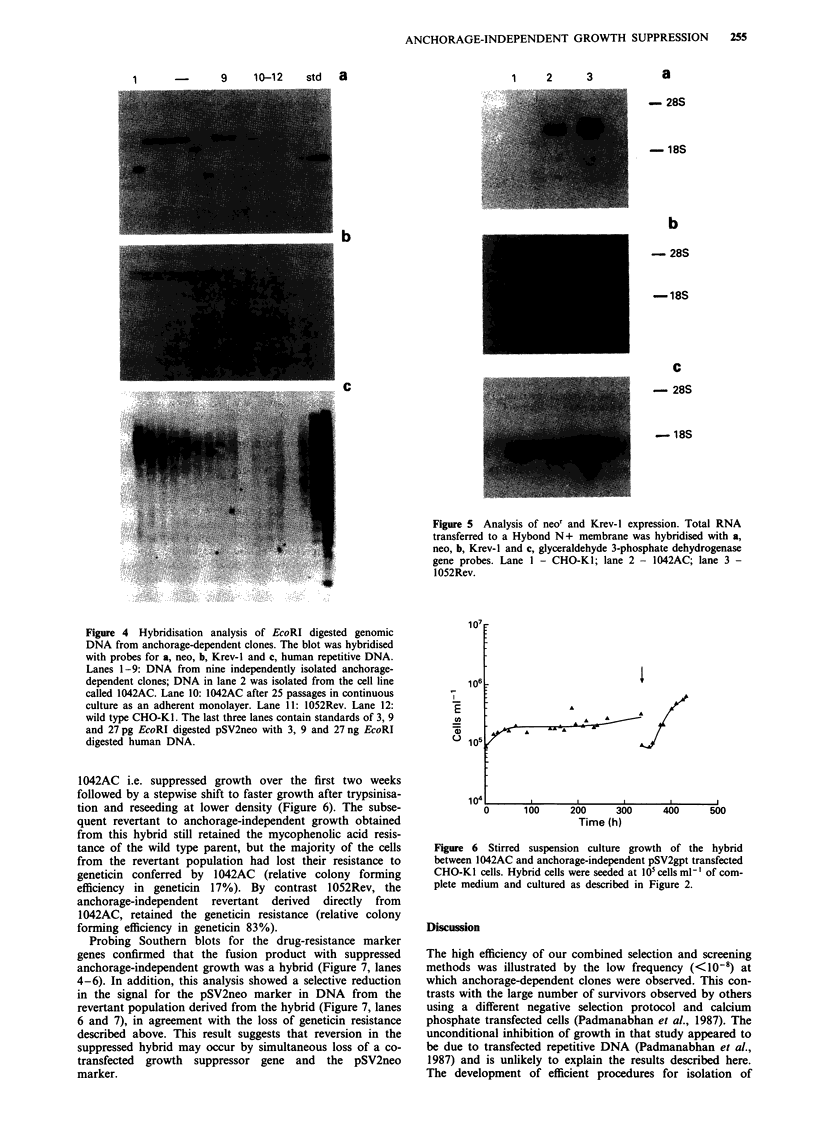

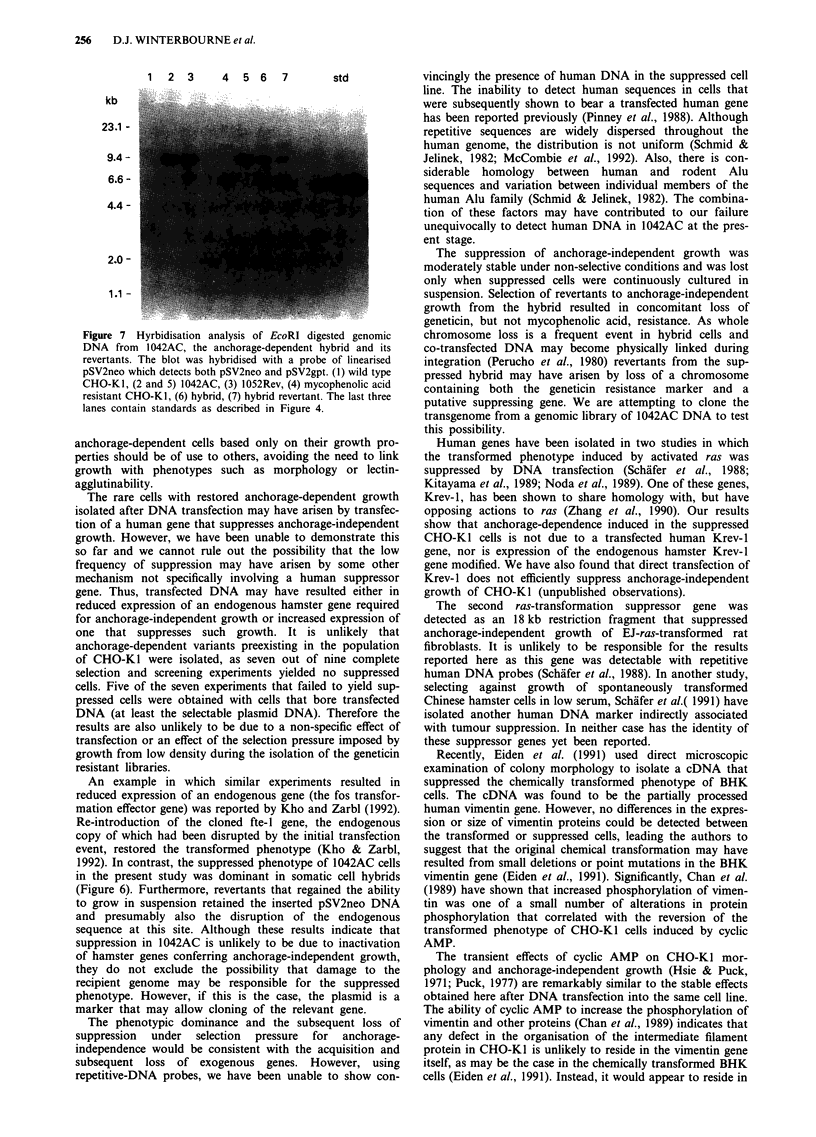

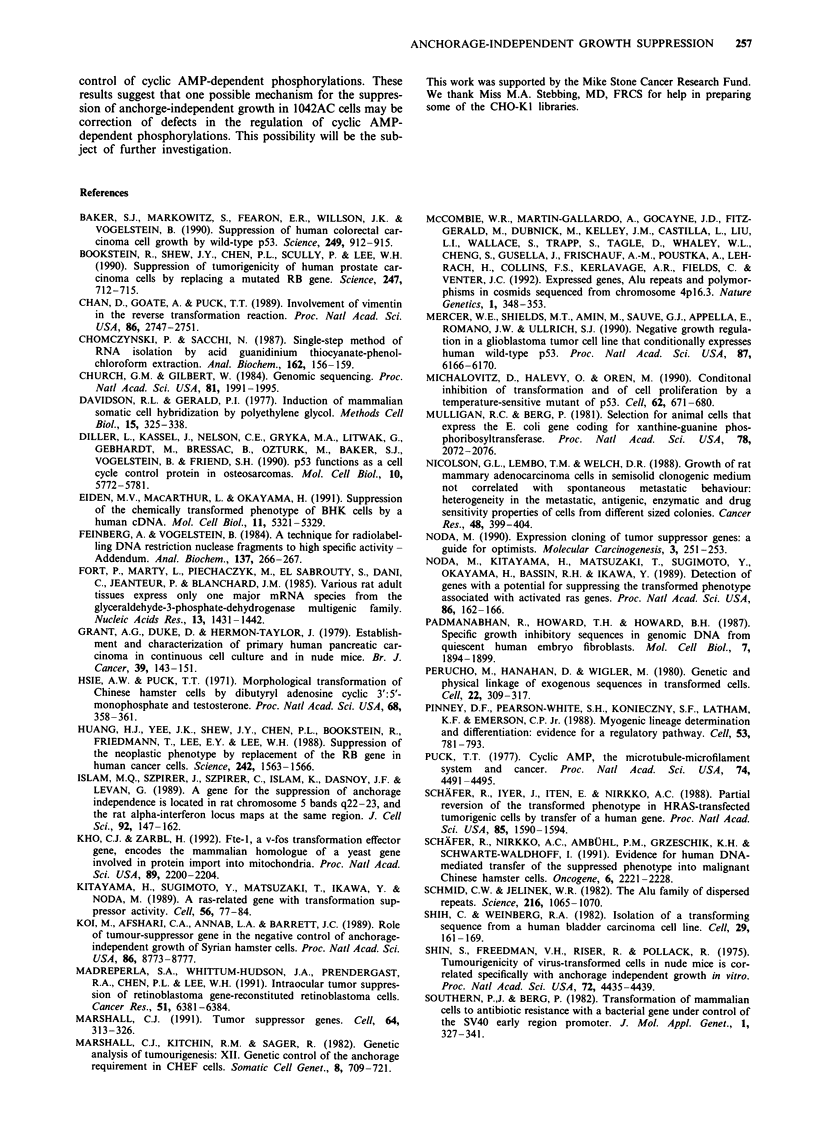

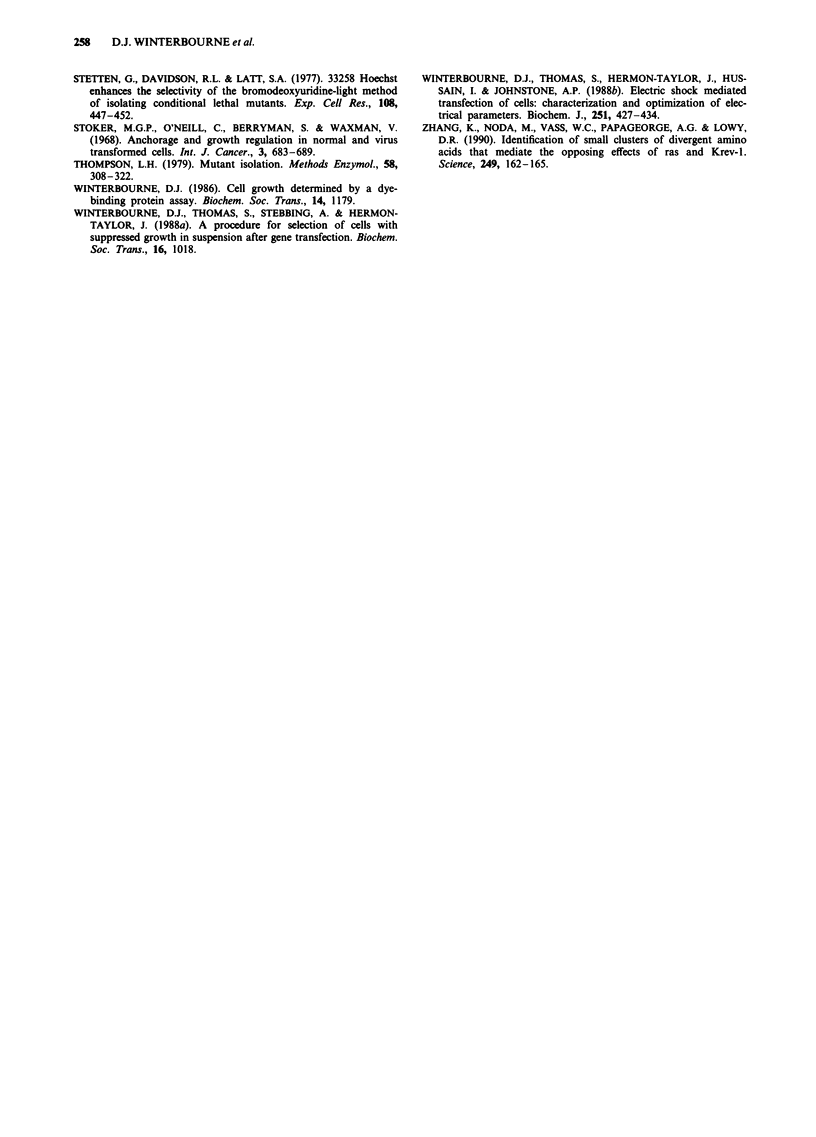


## References

[OCR_00851] Baker S. J., Markowitz S., Fearon E. R., Willson J. K., Vogelstein B. (1990). Suppression of human colorectal carcinoma cell growth by wild-type p53.. Science.

[OCR_00856] Bookstein R., Shew J. Y., Chen P. L., Scully P., Lee W. H. (1990). Suppression of tumorigenicity of human prostate carcinoma cells by replacing a mutated RB gene.. Science.

[OCR_00862] Chan D., Goate A., Puck T. T. (1989). Involvement of vimentin in the reverse transformation reaction.. Proc Natl Acad Sci U S A.

[OCR_00867] Chomczynski P., Sacchi N. (1987). Single-step method of RNA isolation by acid guanidinium thiocyanate-phenol-chloroform extraction.. Anal Biochem.

[OCR_00872] Church G. M., Gilbert W. (1984). Genomic sequencing.. Proc Natl Acad Sci U S A.

[OCR_00876] Davidson R. L., Gerald P. S. (1977). Induction of mammalian somatic cell hybridization by polyethylene glycol.. Methods Cell Biol.

[OCR_00881] Diller L., Kassel J., Nelson C. E., Gryka M. A., Litwak G., Gebhardt M., Bressac B., Ozturk M., Baker S. J., Vogelstein B. (1990). p53 functions as a cell cycle control protein in osteosarcomas.. Mol Cell Biol.

[OCR_00888] Eiden M. V., MacArthur L., Okayama H. (1991). Suppression of the chemically transformed phenotype of BHK cells by a human cDNA.. Mol Cell Biol.

[OCR_00893] Feinberg A. P., Vogelstein B. (1984). "A technique for radiolabeling DNA restriction endonuclease fragments to high specific activity". Addendum.. Anal Biochem.

[OCR_00898] Fort P., Marty L., Piechaczyk M., el Sabrouty S., Dani C., Jeanteur P., Blanchard J. M. (1985). Various rat adult tissues express only one major mRNA species from the glyceraldehyde-3-phosphate-dehydrogenase multigenic family.. Nucleic Acids Res.

[OCR_00905] Grant A. G., Duke D., Hermon-Taylor J. (1979). Establishment and characterization of primary human pancreatic carcinoma in continuous cell culture and in nude mice.. Br J Cancer.

[OCR_00911] Hsie A. W., Puck T. T. (1971). Morphological transformation of Chinese hamster cells by dibutyryl adenosine cyclic 3':5'-monophosphate and testosterone.. Proc Natl Acad Sci U S A.

[OCR_00917] Huang H. J., Yee J. K., Shew J. Y., Chen P. L., Bookstein R., Friedmann T., Lee E. Y., Lee W. H. (1988). Suppression of the neoplastic phenotype by replacement of the RB gene in human cancer cells.. Science.

[OCR_00923] Islam M. Q., Szpirer J., Szpirer C., Islam K., Dasnoy J. F., Levan G. (1989). A gene for the suppression of anchorage independence is located in rat chromosome 5 bands q22-23, and the rat alpha-interferon locus maps at the same region.. J Cell Sci.

[OCR_00930] Kho C. J., Zarbl H. (1992). Fte-1, a v-fos transformation effector gene, encodes the mammalian homologue of a yeast gene involved in protein import into mitochondria.. Proc Natl Acad Sci U S A.

[OCR_00936] Kitayama H., Sugimoto Y., Matsuzaki T., Ikawa Y., Noda M. (1989). A ras-related gene with transformation suppressor activity.. Cell.

[OCR_00941] Koi M., Afshari C. A., Annab L. A., Barrett J. C. (1989). Role of a tumor-suppressor gene in the negative control of anchorage-independent growth of Syrian hamster cells.. Proc Natl Acad Sci U S A.

[OCR_00947] Madreperla S. A., Whittum-Hudson J. A., Prendergast R. A., Chen P. L., Lee W. H. (1991). Intraocular tumor suppression of retinoblastoma gene-reconstituted retinoblastoma cells.. Cancer Res.

[OCR_00957] Marshall C. J., Kitchin R. M., Sager R. (1982). Genetic analysis of tumorigenesis: XII. Genetic control of the anchorage requirement in CHEF cells.. Somatic Cell Genet.

[OCR_00953] Marshall C. J. (1991). Tumor suppressor genes.. Cell.

[OCR_00967] McCombie W. R., Martin-Gallardo A., Gocayne J. D., FitzGerald M., Dubnick M., Kelley J. M., Castilla L., Liu L. I., Wallace S., Trapp S. (1992). Expressed genes, Alu repeats and polymorphisms in cosmids sequenced from chromosome 4p16.3.. Nat Genet.

[OCR_00972] Mercer W. E., Shields M. T., Amin M., Sauve G. J., Appella E., Romano J. W., Ullrich S. J. (1990). Negative growth regulation in a glioblastoma tumor cell line that conditionally expresses human wild-type p53.. Proc Natl Acad Sci U S A.

[OCR_00979] Michalovitz D., Halevy O., Oren M. (1990). Conditional inhibition of transformation and of cell proliferation by a temperature-sensitive mutant of p53.. Cell.

[OCR_00984] Mulligan R. C., Berg P. (1981). Selection for animal cells that express the Escherichia coli gene coding for xanthine-guanine phosphoribosyltransferase.. Proc Natl Acad Sci U S A.

[OCR_00990] Nicolson G. L., Lembo T. M., Welch D. R. (1988). Growth of rat mammary adenocarcinoma cells in semisolid clonogenic medium not correlated with spontaneous metastatic behavior: heterogeneity in the metastatic, antigenic, enzymatic, and drug sensitivity properties of cells from different sized colonies.. Cancer Res.

[OCR_00998] Noda M. (1990). Expression cloning of tumor suppressor genes: a guide for optimists.. Mol Carcinog.

[OCR_01002] Noda M., Kitayama H., Matsuzaki T., Sugimoto Y., Okayama H., Bassin R. H., Ikawa Y. (1989). Detection of genes with a potential for suppressing the transformed phenotype associated with activated ras genes.. Proc Natl Acad Sci U S A.

[OCR_01009] Padmanabhan R., Howard T. H., Howard B. H. (1987). Specific growth inhibitory sequences in genomic DNA from quiescent human embryo fibroblasts.. Mol Cell Biol.

[OCR_01015] Perucho M., Hanahan D., Wigler M. (1980). Genetic and physical linkage of exogenous sequences in transformed cells.. Cell.

[OCR_01020] Pinney D. F., Pearson-White S. H., Konieczny S. F., Latham K. E., Emerson C. P. (1988). Myogenic lineage determination and differentiation: evidence for a regulatory gene pathway.. Cell.

[OCR_01026] Puck T. T. (1977). Cyclic AMP, the microtubule-microfilament system, and cancer.. Proc Natl Acad Sci U S A.

[OCR_01031] Schaefer R., Iyer J., Iten E., Nirkko A. C. (1988). Partial reversion of the transformed phenotype in HRAS-transfected tumorigenic cells by transfer of a human gene.. Proc Natl Acad Sci U S A.

[OCR_01043] Schmid C. W., Jelinek W. R. (1982). The Alu family of dispersed repetitive sequences.. Science.

[OCR_01037] Schäfer R., Nirkko A. C., Ambühl P. M., Grzeschik K. H., Schwarte-Waldhoff I. (1991). Evidence for human DNA-mediated transfer of the suppressed phenotype into malignant Chinese hamster cells.. Oncogene.

[OCR_01047] Shih C., Weinberg R. A. (1982). Isolation of a transforming sequence from a human bladder carcinoma cell line.. Cell.

[OCR_01052] Shin S. I., Freedman V. H., Risser R., Pollack R. (1975). Tumorigenicity of virus-transformed cells in nude mice is correlated specifically with anchorage independent growth in vitro.. Proc Natl Acad Sci U S A.

[OCR_01058] Southern P. J., Berg P. (1982). Transformation of mammalian cells to antibiotic resistance with a bacterial gene under control of the SV40 early region promoter.. J Mol Appl Genet.

[OCR_01066] Stetten G., Davidson R. L., Latt S. A. (1977). 33258 Hoechst enhances the selectivity of the bromodeoxyuridine--light method of isolating conditional lethal mutants.. Exp Cell Res.

[OCR_01072] Stoker M., O'Neill C., Berryman S., Waxman V. (1968). Anchorage and growth regulation in normal and virus-transformed cells.. Int J Cancer.

[OCR_01077] Thompson L. H. (1979). Mutant isolation.. Methods Enzymol.

[OCR_01093] Winterbourne D. J., Thomas S., Hermon-Taylor J., Hussain I., Johnstone A. P. (1988). Electric shock-mediated transfection of cells. Characterization and optimization of electrical parameters.. Biochem J.

[OCR_01097] Zhang K., Noda M., Vass W. C., Papageorge A. G., Lowy D. R. (1990). Identification of small clusters of divergent amino acids that mediate the opposing effects of ras and Krev-1.. Science.

